# Diagnosis and management of an elderly patient with severe tracheomalacia: A case report and review of the literature

**DOI:** 10.3892/etm.2013.1195

**Published:** 2013-07-02

**Authors:** AI-GUI JIANG, XIAO-YAN GAO, HUI-YU LU

**Affiliations:** Department of Respiratory Medicine, Taizhou People’s Hospital, Taizhou, Jiangsu 225300, P.R. China

**Keywords:** tracheomalacia, continuous positive airway pressure, metallic stent

## Abstract

Severe adult tracheomalacia is a dangerous disease that is difficult to manage, particularly at the time of airway infection, and has a high mortality rate. The present study reports the diagnosis and treatment of an elderly patient with severe adult tracheomalacia. In March 2012, the 59-year-old patient presented with progressive dyspnea to the Department of Respiratory Medicine, Taizhou People’s Hospital (Jiangsu, China). Following admission, chest radiography revealed symptoms consistent with chronic obstructive pulmonary disease (COPD) and chest computed tomography (CT) demonstrated an evident stenosis of the tracheal lumen at the end of expiration. Bronchoscopy revealed a 91% reduction in the cross-sectional area of the tracheal lumen at the end of expiration. Following the final diagnosis, the patient was successfully treated with nasal continuous positive airway pressure (CPAP) combined with implantation of a temporary Chinese Li’s metallic stent. These treatment methods appeared to be temporarily effective in alleviating the symptoms of the disease.

## Introduction

Tracheomalacia, a condition characterized by excessive expiratory collapse due to the atrophy and/or reduction of tracheal elastic fibers of the tracheal wall or a reduction in the integrity of tracheal cartilage, is a significant cause of morbidity (1). Softening may occur in part or all of the tracheal cartilage and may even extend beyond the trachea (tracheobronchomalacia). Methods for the treatment of severe tracheomalacia in adults are limited and there is no uniform standard. Surgical treatments, including stent implantation (2,3), tracheostomy tube insertion (1) and external tracheal stabilization (4), have been shown to have a number of therapeutic effects; however, their use requires careful consideration on an individual basis and is generally restricted to patients with localized disease. With regards to medical treatments, the efficacy of corticosteroids in tracheomalacia has not been scientifically proven and the use of continuous positive airway pressure (CPAP) in tracheomalacia is rarely reported (5,6). The present study reports the diagnosis of an elderly patient with severe tracheomalacia and the outcomes of treatment with nasal CPAP combined with implantation of a temporary Chinese Li’s metallic stent devised by Professor Li Qiang from The Second Military Medical University (Shanghai, China; Micro-Tech Co., Ltd., Nanjing, Jiangsu, China).

## Case report

This study was carried out in accordance with the Declaration of Helsinki and approved by the Ethics Committee of Taizhou People’s Hospital, Jiangsu, China. Written informed consent was obtained from the patient. The 59-year-old female patient reported experiences of paroxysmal breathlessness during the previous 5 years and a history of mild repetitive bronchitis, occurring 2–3 times a year in the coldest months. Two months prior to admission, the paroxysmal breathlessness, cough and expectoration had started to occur more frequently, despite treatment with salbutamol sulfate aerosol (Ventolin, 200 μg four times daily), salmeterol/fluticasone (50/500 μg twice daily) and oral moxifloxacin (0.4 g once daily.) As a result of the persistent dyspnea, the patient had a limited ability to carry out normal daily activities. Following admission, physical examinations revealed a body temperature of 37.9ºC, a pulse of 113 beats per min, a respiratory rate of 28 breaths per min and a blood pressure of 140/94 mmHg. The patient exhibited an exhausted appearance and cyanosis of the lips. Respiratory movements and vocal fremitus were equal bilaterally. Expiratory wheezing was heard throughout both lungs and no moist rales were noted. The patient had a regular heart rhythm and no edema was observed in the lower extremities.

**Figure 1:**
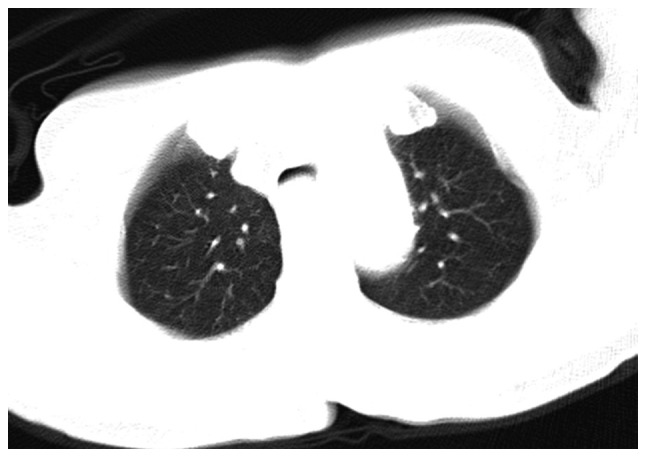
Computed tomography scan revealed evident stenosis of the tracheal lumen at the end of expiration.

**Figure 2:**
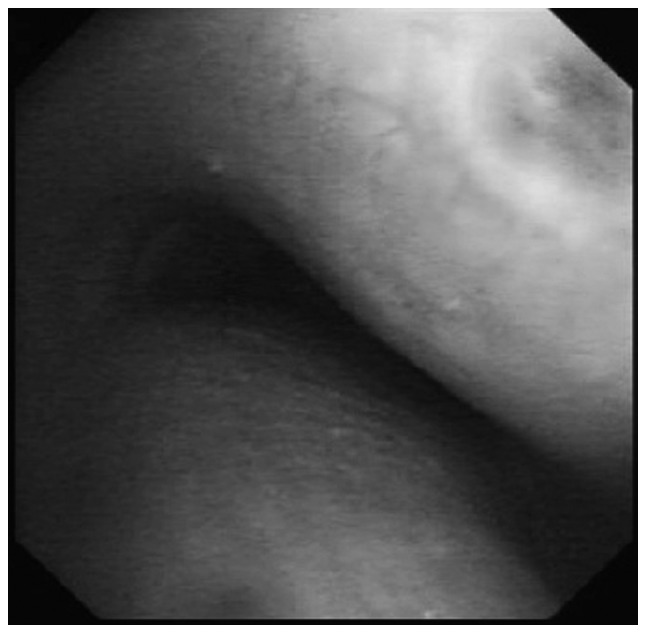
Bronchoscopy revealed a 91% reduction in the cross-sectional area of the tracheal lumen at the end of expiration.

A complete blood test revealed a white cell count of 11.59×10^9^/l and a large white blood cell ratio (percentage of white blood cells that are neutrophils) of 71.2%. A blood gas analysis demonstrated a pH of 7.35, 68 mmHg PaO_2_, 60 mmHg PaCO_2_ and 31 mmol/l HCO_3_^−^ (performed during oxygen inhalation at a low flow rate). A lung function test indicated severe obstructive ventilation disorder with a FEV1/FVC ratio of 37%. Chest radiography showed signs consistent with chronic obstructive pulmonary disease (COPD) and chest computed tomography (CT) demonstrated evident stenosis of the tracheal lumen at the end of expiration (Fig. 1). Bronchoscopy revealed a widening of the membranous part of the trachea, folds, significant airway collapse at the end of expiration and a 91% reduction in the cross-sectional area of the tracheal lumen (Fig. 2).

The patient was diagnosed with adult tracheomalacia and nasal CPAP was administered to improve ventilation. This was combined with antibiotics (intravenous moxifloxacin, 0.4 g once daily), inhaled medication for asthma (budesonide, 4 mg twice daily) and mucolytics (intravenous ambroxol hydrochloride, 120 mg once daily). During CPAP, the pressure level was initially set at 8 cm H_2_O and was then increased in 1 cm H_2_O increments until a level associated with clinical alleviation of the stridor, the greatest reduction in breathing rate and an increase in SaO_2_ was identified. The optimal pressure level for this patient was 12 cm H_2_O. Symptoms, including cough, expectoration, fever and dyspnea, were improved by day 4 following admission and episodes of wheezing and stridor occurred less frequently. On day 16, the patient had improved substantially and was discharged.

Following discharge, the patient was treated with nasal CPAP, the level of which was regulated down to 10 cm H_2_O. For the subsequent 3 months, the patient reported feeling healthy and had an increased level of activity; however, attempts to discontinue the nasal CPAP were accompanied by a marked increase in respiratory distress.

**Figure 3:**
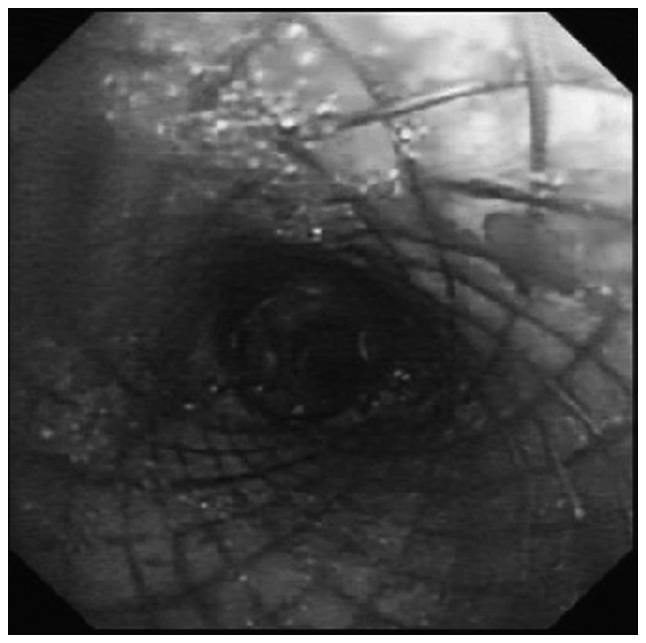
Open trachea following implantation of a a Chinese Li’s metallic stent.

Four months following the initiation of nasal CPAP, breathlessness recurred in the patient and was refractory to an increase in CPAP to 13 cm H_2_O. The patient was readmitted to hospital and a temporary Chinese Li’s metallic stent was implanted using flexible bronchoscopy. The stent was positioned 6 cm below the vocal cords and 2 cm above the eminence, markedly opening the trachea (Fig. 3).

**Figure 4:**
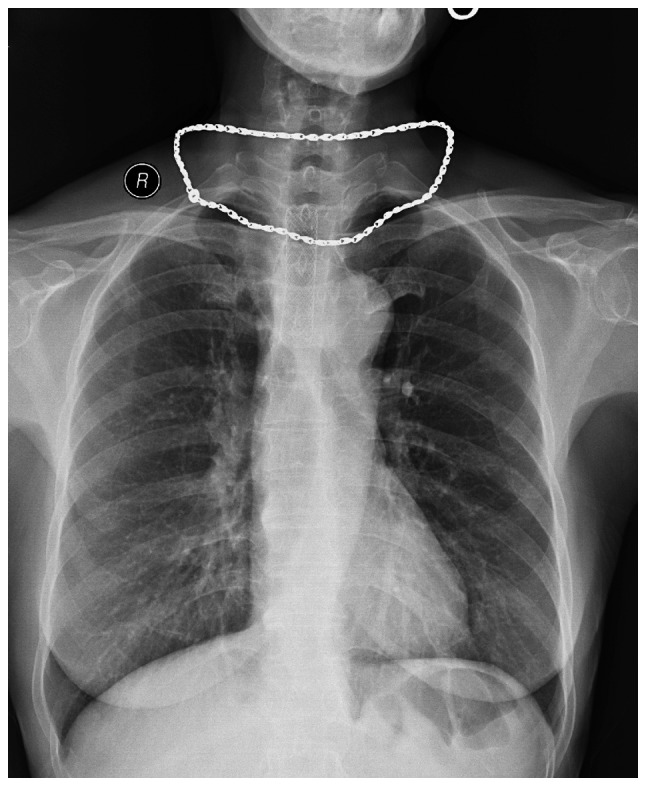
Chest radiography one day after surgery. Images revealed that the positioning and expansion of the metallic stent were successful.

Chest radiography confirmed that the positioning and expansion of the metallic stent were successful (Fig. 4). The patient was also administered nasal CPAP, combined with antibiotics (intravenous moxifloxacin, 0.4 g once daily), inhaled medication for asthma (budesonide, 4 mg twice daily) and mucolytics. Respiratory symptoms, including dyspnea, breathlessness, expectoration and fever, were significantly improved in the patient following placement of the stent.

Three weeks following surgery, the metallic stent was removed since the patient exhibited a marked improvement in breathlessness. Episodes of stridor and respiratory distress were not aggravated by intervention with nasal CPAP combined with salmeterol/fluticasone (50/500 μg twice daily, respectively) and the patient was discharged.

## Discussion

Adult tracheomalacia may be classified into congenital (for example, Mounier-Kuhn syndrome) or acquired forms, including those resulting from chest trauma, tracheostomy, inflammation, chronic irritation, malignancy or mechanical anatomical factors. Softening may affect either part or all of the tracheal cartilage, and may even extend beyond the trachea (tracheobronchomalacia). Although previous studies have shown that adult tracheomalacia occurs in elderly patients and is significantly associated with COPD and smoking, the implications of the coexistence of COPD and tracheomalacia are not fully understood, and the pathological progression of COPD to tracheomalacia has not been clearly demonstrated (5,7). Since it is difficult to differentiate between tracheomalacia and COPD, we were unable to confirm which of the conditions occurred first in the patient.

Although the techniques and criteria for the diagnosis of tracheomalacia are not standardized, thorax CT and bronchoscopy are the preferred methods in previously published studies (8–10). With regards to the degree of airway stenosis, a collapse of <50% is within normal limits, 50–75% is mild, 75–90% is moderate and 91–100% (close proximity of the posterior membrane to the anterior luminal surface) is considered to indicate severe malacia (3). Tracheomalacia patients have no overt symptoms during the initial stages; however, as the disease progresses, they are likely to present with shortness of breath, cough, sputum and other obstructive and infectious symptoms of the respiratory tract. In particular, inspiratory stridor is the distinctive symptom. With increasing levels of awareness and improved diagnosis of the disease, the incidence of reported tracheomalacia has increased significantly (1). Asymptomatic patients require close observation without treatment, and there is no uniform standard treatment for patients with the severe form of the disease. Generally, patients are treated on an individual basis. The efficacy of corticosteroids for the treatment of tracheomalacia has not been scientifically proven. Surgical treatments, including stent implantation (2,3), tracheostomy tube insertion (1) and the external tracheal stabilization technique (4), are generally restricted to patients with localized disease and thus require careful consideration.

CPAP is relatively simple to use, non-invasive and has few side-effects. It exerts its therapeutic effects by increasing tidal volume, which reduces airway collapse and reduces respiratory effort. Consequently, CPAP is the preferred method of treatment in previously published studies (1). There is no uniform standard for adjusting CPAP ventilation to the optimal level. Ferguson and Benoist (6) evaluated expiratory airflow and airway collapse during the acute administration of nasal CPAP in three tracheobronchomalacia patients for whom conventional medical management had failed. It was observed that FVC increased and dynamic airway collapse decreased with increasing levels of CPAP. Davis *et al*(11) reported that the optimal level of CPAP in infants with severe tracheomalacia may be achieved by increasing the lung volume to a level at which the infant is not flow-limited during tidal breathing, without simultaneously increasing the effort of breathing by reducing pulmonary compliance. In the present study, the procedure was initiated with the adjustment of CPAP ventilation to the optimal level. The pressure level was initially set at 8 cm H_2_O and was then progressively increased in 1 cm H_2_O increments and set at a level associated with the clinical alleviation of the stridor, the greatest fall in breathing rate and an increase in SaO_2_. As the procedure has the advantages of rapid evaluation, simple operation, low cost and being non-invasive, it was preferred by the doctors in the Department of Respiratory Medicine, Taizhou People’s Hospital (Taizhou, China) and acceptable to the patient.

To the best of our knowledge, there has been no comparative study between CPAP and bilevel positive airway pressure (BIPAP) in the treatment of adult tracheomalacia. Essouri *et al*(12) reported that non-invasive ventilation using CPAP and BIPAP is associated with a significant and comparable reduction in respiratory effort in infants with severe upper airway obstruction. CPAP ventilation remains the preferred mode over BIPAP, since the inspiratory and expiratory trigger sensitivity of BIPAP mode ventilation is insufficient in patients with a high respiratory rate or small tidal volume, resulting in patient-ventilator asynchrony.

In our experience, a nasal mask is preferable to an oronasal mask for the following reasons: i) Nasal masks are smaller and associated with a reduction in dead space; ii) certain side-effects, including abdominal distention resulting from an ingression of gas to the gastrointestinal tract, are alleviated; iii) the nasal mask is well tolerated and there is reduced gas leakage, particularly in elderly patients with facial bone malformation. Furthermore, since patients may be dependent on CPAP for an extended duration following the diagnosis of tracheomalacia, custom-made nasal masks designed according to the facial bones of the patient may be beneficial.

Intratracheal stent implantation has been used in the treatment of adult tracheomalacia since 1965, primarily in severe cases where traditional treatments have failed (13,14,15). Intratracheal stents are divided into two major types: silicone stents and shape-memory alloy stents. Silicone stents are easily implanted and removed, but have complications including infection, expectoration and the tendency to undergo migration. Furthermore, they require a high-cost surgery involving rigid bronchoscopy and general anesthesia (16). The above-mentioned limitations of silicone stents are overcome by shape-memory alloy stents; however, the use of these in benign airway stenosis is controversial since they are known to be associated with complications, including restenosis, the excessive growth of granulation tissue and stent migration (17). To the best of our knowledge, airway infection is one of the factors leading to the aggravation of adult tracheomalacia and is difficult to control in a short period of time. Chinese Li’s metallic stents not only have the advantages of traditional memory-alloy stents, but may also be adjusted and removed before the hyperplasia of granulation tissue occurs (usually at 3 weeks) due to the unique design of a double recycling line at both ends of the stent. This time-effective advantage allows the earlier application of traditional treatments, including CPAP respiratory support, anti-infection and asthma treatment, which improves the success rate of clinical rescue.

In conclusion, severe adult tracheomalacia is a dangerous disease that is challenging to manage, particularly at the time of airway infection, and has a high mortality rate (8). Nasal CPAP combined with the implantation of a temporary Chinese Li’s metallic stent may be an effective treatment for temporarily alleviating symptoms of the disease.

## References

[1] Carden KA, Boiselle PM, Waltz DA, Ernst A (2005). Tracheomalacia and tracheobronchomalacia in children and adults: an in-depth review. Chest.

[2] Thornton RH, Gordon RL, Kerlan RK (2006). Outcomes of tracheobronchial stent placement for benign disease. Radiology.

[3] Ernst A, Majid A, Feller-Kopman D (2007). Airway stabilization with silicone stents for treating adult tracheobronchomalacia: a prospective observational study. Chest.

[4] Cho JH, Kim H, Kim J (2012). External tracheal stabilization technique for acquired tracheomalacia using a tailored silicone tube. Ann Thorac Surg.

[5] Kandaswamy C, Balasubramanian V (2009). Review of adult tracheomalacia and its relationship with chronic obstructive pulmonary disease. Curr Opin Pulm Med.

[6] Ferguson GT, Benoist J (1993). Nasal continuous positive airway pressure in the treatment of tracheobronchomalacia. Am Rev Respir Dis.

[7] Murgu SD, Colt HG (2006). Treatment of adult tracheobronchomalacia and excessive dynamic airway collapse : an update. Treat Respir Med.

[8] Jiang A, Lu H (2012). Early diagnosis and management of tracheomalacia with invasive bronchopulmonary aspergillosis in an adult. Braz J Infect Dis.

[9] Gilkeson RC, Ciancibello LM, Hejal RB, Montenegro HD, Lange P (2001). Tracheobronchomalacia: dynamic airway evaluation with multidetector CT. AJR Am J Roentgenol.

[10] Aquino SL, Shepard JA, Ginns LC (2001). Acquired tracheomalacia: detection by expiratory CT scan. J Comput Assist Tomogr.

[11] Davis S, Jones M, Kisling J, Angelicchio C, Tepper RS (1998). Effect of continuous positive airway pressure on forced expiratory flows in infants with tracheomalacia. Am J Respir Crit Care Med.

[12] Essouri S, Nicot F, Clément A (2005). Noninvasive positive pressure ventilation in infants with upper airway obstruction: comparison of continuous and bilevel positive pressure. Intensive Care Med.

[13] Sommer D, Forte V (2000). Advances in the management of major airway collapse: the use of airway stents. Otolaryngol Clin North Am.

[14] Casiano RR, Numa WA, Nurko YJ (2000). Efficacy of transoral intraluminal Wallstents for tracheal stenosis or tracheomalacia. Laryngoscope.

[15] Ernst A, Odell DD, Michaud G, Majid A, Herth FF, Gangadharan SP (2011). Central airway stabilization for tracheobronchomalacia improves quality of life in patients with COPD. Chest.

[16] Murgu SD, Colt HG (2007). Complications of silicone stent insertion in patients with expiratory central airway collapse. Ann Thorac Surg.

[17] Chen W, Ruan Y (2012). Late complications of nickel-titanium alloy stent in tracheal stenosis. Laryngoscope.

